# Role of celebrity endorsement in promoting employees’ organization identification: A brand-based perspective

**DOI:** 10.3389/fpsyg.2022.910375

**Published:** 2022-09-08

**Authors:** Muhammad Abdullah, Sidra Ghazanfar, Rakhshan Ummar, Rizwan Shabbir

**Affiliations:** ^1^Department of Management Sciences, Khwaja Fareed University of Engineering and Information Technology, Rahim Yar Khan, Pakistan; ^2^Department of Management Sciences, National University of Modern Languages, Islamabad, Pakistan; ^3^Lyallpur Business School, Government College University, Faisalabad, Pakistan

**Keywords:** organization identification, brand orientation, celebrity endorsement effectiveness, employee pride, social identity theory

## Abstract

Celebrity endorsement has been used for decades to promote products to consumers. As employees are one of the primary stakeholders and are known as second consumers, their concerns about celebrity endorsement effectiveness and pride need attention for building their identification with an organization. This study investigated the internal branding process by examining employees’ brand orientation, celebrity-organization value congruence, and the accuracy of employee portrayal. Data are collected from a leading multinational bank in Pakistan through a structured questionnaire. The results of the study showed that when employees felt celebrity endorsement matched organizational values, the celebrity successfully portrayed actual corporate values. Thus, employees believed that endorsement effectively gained consumers’ attention and built a strong corporate image. The study affirmed that employees’ sense of pride toward their organization motivates them to identify with it. Furthermore, the results showed that value congruence mediates the relationship between brand orientation and endorsement effectiveness, while pride mediates the relationship between endorsement effectiveness and organization identification. Service organizations could use brand orientation to gain accurate employee portrayal that revives their pride and attachment with the organization and enhances corporate identification. The future directions and limitations are discussed.

## Introduction

In marketing, celebrity endorsement is a very common phenomenon in the twenty-first century. Knoll and Matthes (2017) concluded that almost celebrities have appeared in every fifth advertisement. The study of Carlson et al. (2020) suggested that agencies have spent 10% of advertisement costs on promoting endorsements. In contrast, few multinational organizations have spent more than 25% of their promotion on endorsers. A celebrity is a personality who gains public appreciation and recognition from the name of the organization, whom they are endorsing for the purpose of promotion (McCracken, 1989).

Celebrities enjoy fame and popularity, influencing the endorsed brand’s image. The celebrity portrays a clear image of an organization to consumers. Brand marketing by a celebrity or a famous personality develops attractive appeal, gains more attention, and high recall (Davies and Slater, 2015). Companies use celebrities to create a distinguished position in the market and to build a positive brand image (Ranjbarian et al., 2010), which shapes a positive consumer attitude and a unique brand personality/organization (Thomson, 2006). Marketing managers and advertisers use endorsement strategies such as celebrity endorsement for their organizations because they understand that consumer attitude toward a celebrity also transfers to the organization (Choi and Rifon, 2012). Therefore, marketers and advertisers hire an appropriate celebrity because the endorsement by consumers’ accepted celebrity enhances consumers’ positive attitude (Surana, 2008). The work of Schimmelpfennig (2018) also concluded that positive association of celebrity-brand congruence with brand evaluation played a role in designing advocacy and engagement.

Organizational identification is considered a strategic asset for creating creditability among stakeholders, and it leads marketing managers to gain a competitive advantage by fabricating a dynamic corporate environment (Melewar, 2003). Organizational identification is the prime motivation for employees to understand better corporate goals, vision, and culture, which present pride, and image (Downey, 1987). An organization with good corporate identification attracts executives with exceptional skills that provide admirable financial outcomes (Melewar and Saunders, 1998). With a definite identification, it is easier to portray organizational capabilities, differentiation strategies, and product/service diversification (Lippincott and Margulies, 1988). Moreover, the consumer perspective presented that product quality, brand image, and loyalty are linked with organizational identification that portrays brand orientation for external and internal customers. Thus, organization identification is a step toward producing a corporate brand that brings a sense of community among stakeholders (Markwick and Fill, 1997).

Corporate marketing explains the coordination of a brand based on its organizational culture and knowledge (Balmer, 2013). It presents a unique strategic organizational approach (Illia and Balmer, 2012; Balmer, 2017) that constitutes identification-based corporate orientation. Corporate marketing describes an organizational structure that allows all stakeholders to exchange their values and beliefs mutually similar to the marketing orientation, which comprises internal/external communication mediums like TVCs, publicity, public relations, and endorsements (Balmer et al., 2020). Corporate branding is an effective tool for managers to build emotional ownership among stakeholders, especially by using celebrities as brand ambassadors/endorsers.

Employees of the organization are also part of that audience influenced by celebrities. Berry (1980) and Papasolomou and Vrontis (2006) highlighted that employees are internal customers, and their support is essential for successful marketing programs. Employees also interpret, judge, and react to the marketing communication strategies of their organization (Gilly and Wolfinbarger, 1998; Scott and Lane, 2000), which affects their satisfaction (Shostack, 1987) and supports them in identifying with their organization (Khan and Stanton, 2010; Farrelly et al., 2012; Hofer and Grohs, 2018). Employee pride is always a priority for reputable organizations.

Recent studies of organizational behavior suggested pride as an emerging area, and academic researchers investigated their relationships with the workplace (Ellington and Wilson, 2017; Kraemer et al., 2020). Various marketing techniques are used for internal marketing, which helps to shape behaviors for the achievement of organizational goals (Rafiq and Ahmed, 2000; Hartline and Bejou, 2004) and guide employees to live the brand image of their organization (Miles and Mangold, 2004). Ashforth and Mael (1989) and Dutton et al. (1994) studied the effects of new media advertising on employees’ attitudes, Celsi and Gilly (2010) studied the effects of advertising, and Hofer and Grohs (2018) studied the effects of sponsorship toward organization identification and performance outcomes.

Corporate marketing demands those marketing strategies that are customer-oriented, which deliver valuable identification of organization and employees in society. The study by Carlson et al. (2020) argued that unproductive endorsement attracts customers toward the product attachment rather than building customer association/engagement. However, customer-endorser affiliation built a sense of community for internal and external customers. In the service sector, the association of celebrity endorsement plays a central role in generating identification because “people” are one of the key promotional mix elements. The study proposed that optioning relevant endorsers could play a transforming role in building employee pride and organizational identification.

Moreover, the direct association of celebrity endorsers generates customer knowledge and recognition of its expertise and product-endorser fit. A competitive organization consists of attributes such as marketing strategy, corporate culture, and behavior, and these aspects build identity. Nowadays, corporate identity is getting attention as a strategic goal in disciples like marketing and behavioral sciences (Melewar, 2003). To experience consistency for a corporate brand, employee commitment and behavior indicate brand values (Lohndorf and Diamantopoulos, 2014; Piehler et al., 2016), pride, brand promise (Piha and Avlonitis, 2018), and desired level of brand identity (Harris and De Chernatony, 2001). Morhart et al. (2009) concluded that employees are vital for building organizational identity because any brand-branding intention needs all stakeholders to follow marketing mix strategies (Lohndorf and Diamantopoulos, 2014). While promoting product/service, frontline employees deliver the organizational promise that expresses their sense of belonging with the organization (Boukis et al., 2021), characterized as employee pride. Employee pride is considered a psychological tendency toward an organization when an employee believes that his/her performance exceeds expectations (Kraemer et al., 2020). The value of employee pride provides value-based appraisal and generates organizational identification (Thomas et al., 2018).

The study used stimulus–organism–response (SOR) theory to mark the research gap by investigating celebrity value congruence, employee pride, and organizational identification of employees. From an organizational perspective, assumptions of social identity theory (SIT) are employed to interpret the psychological mechanism of identifying an organization. This theory states that individuals see groups as a reference point for gathering/sharing information about others/themselves (Tyler et al., 1996). Thus, employees adopt the social status of their organization and present their pride (Tyler, 1999) by following corporate values/beliefs, such as advocating celebrity promotion/endorsement. The employees’ perspective suggested that an individual identification with the corporate world provides prestigious standings because being a part of that community brought pride and self-enhancement among stakeholders. Pratt (1998) focused on insights into social identification principles by investigating an individual’s relationship with his/her social group; in this case, it is brand orientation with employee identification.

Organizations benefit from encouraging identification among employees, as their identification ensures that employees will prefer their organizational interests (Cheney, 1983). Therefore, the growth of any organization is accredited to its in-depth orientation (Wright et al., 1995), and it might be linked with organizational commitment, competitive advantage, and overall organizational performance (Urde, 1994; Wong and Merrilees, 2007; Baumgarth and Schmidt, 2010). Moreover, it enhances employees’ identification and attachment with their organization (Foster et al., 2010).

## Theoretical support and conceptual framework

The conceptual framework of this study is based on the cognitive psychological theory of SOR (Zimmerman, 2012). A stimulus is an object which induces an effect on an individual. Stimulus is the environmental cues that influence consumers’ feelings and can change a consumer’s overall behavior (Zimmerman, 2012). The SOR theory supports the effect of value-congruent endorsement on employees’ attitudes and behavior. The research framework hypothesizes that stimuli or environmental cues (celebrity-organization value congruence) affect the organism (employees’ emotional feelings), which produces effects (employees’ identification with their organization) (Rajaguru, 2014). Therefore, the study proposed that celebrity-organization value congruence leads to endorsement-linked pride, which enhances employees’ organization identification. Many studies have characterized different stimuli and organisms as a predictor of employee brand identification (Smidts et al., 2001; Mignonac et al., 2006; Bartels et al., 2007; Celsi and Gilly, 2010) based on the SOR model.

Based on organizational identification theory (Mael and Ashforth, 1992; Dutton et al., 1994), the study proposed that internal branding and employee orientation enhance employees’ identification with their organization. Internal branding processes aiming at increasing awareness and commitment of the employees toward their organization are essential to successfully implementing the organization’s policies (Foster et al., 2010). The growth of any organization is accredited to its thorough orientation (Wright et al., 1995), which might be linked with organizational commitment, competitive advantage, profitability, and overall organizational performance, and success (Urde, 1994), and employee identification and attachment with the organization (Baumgarth and Schmidt, 2010).

Internal marketing theories clearly state that internal relations and interactions are important for employees’ involvement, shaping their positive attitudes and motivating them to implement profitable corporate programs (Ahmed et al., 2003; Saleem and Iglesias, 2016). Brand-oriented culture and philosophy guide an organization toward corporate goals (Balmer and Balmer, 2013). Brand equity is believed to be created and protected through a brand-oriented mindset (M’zungu et al., 2010). Some studies have confirmed the role of brand orientation and internal branding on performance outcomes, but limited attempts have been made on their effects on employees’ behavior (Punjaisri and Wilson, 2007; De Chernatony et al., 2010).

Brand orientation facilitates long-term organizational survival. In such organizations, top management executives focus (Wong and Merrilees, 2005) on their brands and align organization strategies with brand strategies (Aaker, 1996), which result in higher brand performance (Gromark and Melin, 2011). Brand orientation is a system in which all the organization’s processes work together to create, develop, and protect an organization’s brand identity (Urde, 1999). Furthermore, it is an organizational culture that promotes the dominant role of a brand in an organization’s strategies and decisions (Wong and Merrilees, 2007; Baumgarth and Schmidt, 2010). It is an inside-out identity view that presents the brand as a part of an organization’s strategies and decisions (Urde et al., 2013).

Brand orientation acts as a mindset that contributes to organizational vision, mission, and values (Urde, 1999; Urde et al., 2013) and supports employees to live their brand (Ind, 2004), which enhances their brand identity and they grow as a strategic resource for an organization (Urde, 1999; De Chernatony et al., 2010; Wilden et al., 2010). Brand orientation facilitates organizational-wide commitment. Commitment and understanding of organizational values support employees to accept the external marketing communication, strengthening their existing beliefs and attitudes toward organizational values (Chakravarti et al., 1997). Hence, the employees who better understand their organizational values positively evaluate the celebrity endorser.

Many marketers and advertisers believe selecting the right celebrity is essential because consumers look for the fit between the celebrity and the organization/product (Choi and Rifon, 2012). A good congruence between the celebrity and organization/product effectively generates a positive endorsement/advertisement evaluation and increases the endorser’s believability (Davies and Slater, 2015). Previous studies found that employees expect value congruence between their organization and marketing communication (Gilly and Wolfinbarger, 1998; Celsi and Gilly, 2010). As members of an organization, employees have detailed knowledge and use it to assess the congruence between the celebrity’s and their organization’s values. Employees believe that as the celebrity is portraying the values of an organization, he/she should be matched with the organizational values (Choi and Rifon, 2012). Hence,

H1a: Brand orientation positively impacts employees’ perception of celebrity-organization value congruence.

Understanding their shared values within an organization helps employees assess the match between the celebrity portrayal values and the employees’ actual characteristics and behavior (Hogg and Turner, 1985). Tajfel and Turner (1982) revealed that people take self-categorization as a reference point to study the similarities between themselves and other members of a group. Therefore, employees evaluate the employee portrayal accuracy by endorser celebrity.

H1b: Brand orientation positively impacts employees’ perception of employee portrayal accuracy.

The celebrity-brand/product congruence plays an important role in deciding the effectiveness of a celebrity endorsement campaign (Hoogland et al., 2007). The celebrity casts a positive image on consumers, making endorsement more persuasive, thus increasing the organization’s attractiveness. A strong link between the product/brand and celebrity is effective for attaining positive evaluations toward advertisement, which ultimately influences the endorser’s believability (i.e., he will successfully portray the actual organization’s and employee’s value) and advertisement effectiveness (Davies and Slater, 2015). Literature suggests that value-congruent advertisement develops and enhances motivational characteristics toward portrayed values (Verplanken and Holland, 2002), which ultimately motivates employees to support the image of their organization portrayed by the endorser. The literature also supports that individuals who perceive similarities between external communication and existing beliefs and values show more assimilation to organizational communication (Sherif et al., 1965; Meyers and Sternthal, 1993).

H2: (a) Celebrity-organization value congruence and (b) employee portrayal accuracy positively impact the perception of endorsement effectiveness.

Endorser value congruence is the likeness of an employee’s values and those values portrayed by the celebrity. Endorser gives statements about organizational values, persuading employees to invoke their values and compare them to celebrity portrayed values.

H3: Celebrity-organization value congruence positively impacts employee portrayal accuracy.

Organizational behavior scholars admit the influence of external organizational images on employees’ attitudes (Ashforth and Mael, 1989; Dutton et al., 1994). When employees feel that the endorsement has effectively gained consumer attention and positively influenced the organization’s image, they “bask in the reflected glory” of the success of their organization (Cialdini et al., 1976). So we propose that:

H4: Celebrity Endorsement effectiveness positively influences employee’s pride.

**FIGURE 1:**
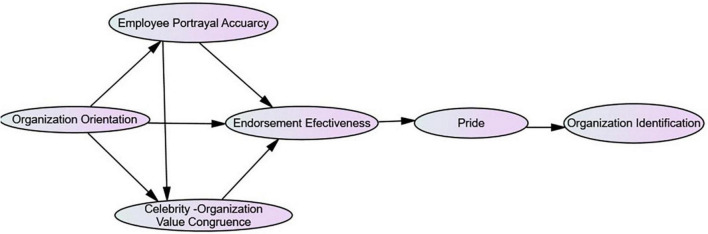
Conceptual framework.

Michie (2009) suggested that pride promotes behaviors that comply with an organization’s social norms. It encourages employees to identify strongly with their employer as they feel honored to be linked with an organization that is publically appreciated (Dutton et al., 1994). The brand-equity relationship has been found important for gaining a consuming response and emphasizing their role in celebrity endorsement (Albert et al., 2017). The positive impression of a celebrity using a service/product is transferred to employees/customers, who also adopt the usage pattern of their beloved celebrity to build an endorser-brand relationship (Carlson et al., 2020). In consumer studies, consumer-endorser identification has been achieved through the effectiveness of the celebrity identification process (Kamins et al., 1989), which means that recognizable celebrities impact organization identification process. Similarly, SIT approach states that employees use the social status of their organization to guide them in estimating their self-worth (Tyler, 1999). Thus, individuals try to identify with organizations, which have prestigious standings, because membership of a prestigious organization increases their self-esteem and fulfills the need of self-enhancement (Figure 1).

H5: Employee pride positively influences employee organizational identification.

## Participants and procedures

To collect data from Habib Bank Limited (a leading commercial bank in Pakistan), we contacted the headquarters of HBL to request their support in data collection from their employees. Habib Bank Limited is a commercial bank in Pakistan with 1,650 branches all over Pakistan. Data were collected from 150 employees from HBL headquarters (Ndubisi et al., 2014). The sample-to-item ratio is generally recommended for exploratory factor analysis, which uses the number of items to decide the sample size in a study. The ratio between the item and the sample size should not be less than 5–1 (Gorsuch, 1983). Hair et al. (2018) suggested a sample-to-variable ratio to decide on sample size in a study. It is suggested that the minimum observation-to-variable ratio should be 5:1, but ratios of 15:1 or 20:1 are preferred.

The results of the demographics showed that 73% of respondents were male. More than 59% of the respondents were under the age of 40, showing that most of the employees are young and have been working with the organization for more than 10 years. An invitation (through email) to an online survey was sent to different employees working at the headquarters of HBL. Once they agreed to fill out the survey, the questionnaire was shared with them. The participants first responded to the items of brand orientation. After watching the HBL endorsement advertisement by famous cricketer Shaheen Shah Afridi, they evaluated celebrity-organization value congruence, employee portrayal accuracy, endorsement effectiveness, pride, and organization identification, which they feel resulted from watching the endorsement advertisement.

### Measures

The constructs used in the study are accounted as “situated” variables because their responses were obtained as a reaction to the cue, an endorsement advertisement in our study that triggered temporary “situated” emotions such as pride and organization identification. The construct “brand orientation” was assessed on five items adopted from the study of Baumgarth and Schmidt (2010). “Value congruence” was assessed on three items adapted from Choi and Rifon (2012). Similarly, “pride” was assessed on three items, while “employee portrayal accuracy,” “endorsement effectiveness, and organization identification” were assessed on four items/each adopted from Celsi and Gilly (2010). The adopted items are mentioned in Table 1. After responding to questions related to the constructs, respondents answered the questions, including participants’ demographic information, such as gender, age, and years of experience in the organization. The study employed structural equation modeling (SEM) for data analysis. SEM is a multivariate technique used to evaluate multivariate causal relationships among variables. SEM is a multivariate statistical tool used to analyze structural relationships and structural links between latent and measured variables.

**TABLE 1:** Items, factor loadings, and reliability.

Construct	Factor loadings	Reliability
**Brand orientation**		0.82
In our company, we have a clear idea of what our brand stands for; brand identity and brand promise are well defined	0.92	
We use all our marketing activities to develop our brand and enhance its strength	0.96	
We recognize our brand as a valuable asset and strategic resource, which we continually develop and protect in the best possible way.	0.87	
Brand equity (or brand strength) is a control factor in our company.	0.93	
All business decisions are evaluated with respect to their impact on the brand	0.77	
**Value congruence**		0.87
I think that Shaheen Shah Afridi promoting HBL is a good fit	0.91	
I think that Shaheen Shah Afridi is compatible with the image of HBL	0.84	
I think that the values of Shaheen Shah Afridi are congruent with the values of HBL	0.83	
**Employee portrayal accuracy**		0.88
Employees are accurately portrayed by the celebrity	0.77	
Employees has shown by the celebrity as they really are	0.79	
I believe many employees in the organization are similar to those portrayed by the celebrity	0.92	
Employees can live up to the image shown by celebrity	0.68	
**Endorsement effectiveness**		0.89
I believe the endorsement by celebrity will increase sales of HBL	0.94	
I believe that the endorser will be well liked by customers	0.96	
The endorsement by this celebrity effectively raise the visibility of HBL	0.89	
The endorsement by celebrity is effective	0.90	
**Pride**		0.85
The celebrity endorser make me proud of HBL	0.88	
Seeing celebrity endorsing HBL makes me feel good about HBL	0.92	
I enjoy telling others about the celebrity endorser	0.91	
**Organization identification**		0.83
I am proud to tell others I am part of HBL	0.92	
I care about the fate of HBL	0.85	
HBL successes is my successes.	0.75	
I am willing to put in a great deal of effort beyond that normally expected in order to help HBL be successful	0.86	

## Results

The study has used confirmatory factor analysis (CFA) to estimate the discriminant validity, convergent, and reliability of the research model. AMOS was used to estimate the maximum likelihood of the measurement model. The model showed acceptable fit indices: χ^2^ = 257.012 with 1.147 CMIN/DF. The values of TLI, CFI, IFI, RFI, and NFI lie between 0.91 and 0.97, and the value of the root mean square error of approximation (RMSEA) was 0.04. The GFI, CFI, and AGFI values are above 0.90 in the measurement model, indicating model fitness (Browne and Cudeck, 1993). Goodness-of-fit indices showed that the data fit thus supports construct validity. CFA was used to estimate the convergent validity of the constructs (Churchill, 1979). Convergent validity is supposed to occur if the pattern coefficient of the indicator exceeds 0.50 and the overall model has acceptable fit indices (Bagozzi et al., 1991).

Cronbach’s alpha coefficient value was used to estimate the internal consistency, and all the values of Cronbach’s alpha were found to be greater than the proposed threshold point of 0.70 (Nunnally, 1978). The Cronbach’s alpha value for construct brand orientation was measured at about 0.82, celebrity-organization value congruence was 0.87, employee portrayal accuracy was 0.88, endorsement effectiveness was 0.89, pride was 0.85, and organization identification was 0.83. These are significantly higher than the cutoff point of 0.70, confirming the constructs’ reliability (Nunnally, 1978; see Table 1). Factor loadings were found significant at the 0.01 level, and loadings above 0.40 were retained for analysis (see Table 1).

The average variance extracted (AVE) was estimated to study the discriminant validity. As Fornell and Larcker (1981) suggested, discriminant validity is supposed to occur if the AVE of a construct is greater than the correlation value with other constructs. All the AVEs values are larger than the threshold level of 0.50. Discriminant validity has been confirmed by following Fornell–Larcker’s criterion that all the square roots of AVEs are greater than their respective correlation values (Hew and Sharifah, 2017; see Table 2).

**TABLE 2:** Discriminant validity, convergent validity, and R-square of O.I.

	Orientation	VC	EPA	EE	Pride	OI	AVE	CR	R
Orientation	0.83						0.690	0.991	
V.C.	0.28	0.92					0.086	0.089	
EPA	0.38	0.38	0.76				0.951	0.803	
E.E.	0.03	0.24	0.20	0.84			0.718	0.929	
Pride	0.16	0.28	0.26	0.44	0.88		0.774	0.916	
O.I.	0.09	0.24	0.00	0.20	0.38	0.81	0.717	0.912	0.31

Diagonal values are the sq-root of AVE; VC, value congruence; EPA, employee portrayal accuracy; EE, endorsement effectiveness; OI, organization identification; AVE, average variance explained; CR, composite reliability.

The results showed a significant relationship between brand orientation and organization-celebrity value congruence, hence supporting H1a (β = 0.56, *p* < 0.05). The relationship between brand orientation and employee portrayal accuracy is also significant, which supports H1b (β = 0.58, *p* < 0.05). Hence, the study supports the idea that brand orientation shapes employees attitude toward organization policies and decisions. Brand orientation also indirectly affects endorsement effectiveness (β = 0.05). Brand orientation does not directly affect endorsement effectiveness, so organization-celebrity value congruence was found to mediate the relationship between brand orientation and endorsement effectiveness. The results further showed a significant relationship of organization-celebrity value congruence with endorsement effectiveness and with employee portrayal accuracy, hence supporting H2a (β = 0.32, *p* < 0.5) and H3 (β = 0.42, *p* < 0.5), while employee portrayal accuracy was found to be insignificant with endorsement effectiveness, rejecting H2b. However, employee portrayal accuracy partially mediates the relationship between organization-celebrity value congruence and endorsement effectiveness. Endorsement effectiveness significantly correlates with pride (β = 0.76, *p* < 0.001) and supports H4. The relationship between pride and organization identification is also significant, supporting H5 (β = 0.37, *p* < 0.001). The relationship between endorsement effectiveness and organization identification is fully mediated by pride, as there was no direct effect of endorsement effectiveness on organization identification. Hence, the study supports the idea that pride associated with an organization’s activities and policies could lead to employees’ identification with the organization.

## Discussion

The goal was to investigate whether brand orientation enhances employees’ organization identification or not. The study supports that internal branding influences employees’ organization identification. Thus, brand orientation encourages supportive organizational behaviors that motivate employees in the workplace. The study provides empirical evidence of the proposed association between brand orientation and employees’ identification with their organization, hence confirming previous literature on the consequences of internal branding (Ahmed and Rafiq, 1999; Punjaisri et al., 2008; Hirvonen et al., 2013; Biedenbach and Manzhynski, 2016). Brand orientation is an organizational culture that promotes the dominant role of a brand in an organization’s strategies and decisions (Wong and Merrilees, 2007; Baumgarth and Schmidt, 2010). The results showed that brand orientation could help employees to positively associate with organizations’ actions and take decisions accordingly, which is in line with the conclusion provided by M’zungu et al. (2010) and Balmer and Balmer (2013). Therefore, it encourages employees to associate with organizations’ celebrity endorsers.

The study confirmed that brand orientation positively impacts employees’ perceptions of employee portrayal accuracy and organization-celebrity value congruence, which is in line with the results of Matanda and Ndubisi (2013), who confirmed the impact of internal customer orientation on person-organization fit. Furthermore, the study also supported the findings of prior research stating that organizational competencies can promote employees’ commitment (Ahmed et al., 2003) because brand orientation shares a strong covenant relation with its stakeholders (including employees) (Balmer, 1998, 2001; Balmer and Gray, 2003). It promotes a culture of shared beliefs, behaviors, and expectations (Chatman and O’Reilly, 2016), shaping employees’ attitudes and behaviors (Baumgarth and Schmidt, 2010) toward celebrity endorsers and promoting organizational acceptability’ values portrayed by the celebrity.

Brand orientation promotes brand-supportive behavior that ensures that employees shape their behavior in correspondence with the brand identity and its values (Baumgarth and Schmidt, 2010). When employees come across a brand image of their organization, they get motivated to play their role as a stakeholder and attempt to relate it to their own identity (Gregory, 2007). Therefore, the employees who work with brand-oriented organizations feel a fit between celebrity-organization values.

Moreover, employees’ perception of celebrity-organization values congruence enhances the perception of endorsement effectiveness. These results are in line with the finding of Celsi and Gilly (2010) and Choi and Rifon (2012), who supported that selection of a favorable celebrity is essential because consumers look for the fit between the celebrity and the organization/product (Davies and Slater, 2015). Therefore, the study concluded that good congruence between the celebrity and organization/product effectively generates positive endorsement/advertisement evaluation and increases endorsers’ believability. The work of Meyers and Sternthal (1993) also suggested that employees who perceive similarities between external communication and existing beliefs and values show more attachment and advocate unique organization identification. However, this study does not support the role of employee portrayal accuracy in endorsement effectiveness, which is not in line with the previous literature (Celsi and Gilly, 2010) because employees perceive that puffery is part of an advertisement and integrated marketing communication (IMC) is designed for customers (James and Alman, 1996; Gilly and Wolfinbarger, 1998). From the employee perspective, employees’ portrayal accuracy has no impact on endorsement effectiveness, as they understand organizational culture and philosophy.

The cognitive theory of emotion proposes that emotions are developed in response to an activity (effective endorsement) which is judged to its effects on one’s wellbeing (such as organization success) (Michie, 2009). The results showed that effective endorsement brought employee pride, which is in line with the findings of Celsi and Gilly (2010). The effective endorsement gives a sense of pride to employees as it successfully promotes a positive image of the organization among consumers and society because of its position in society (Simon, 1995). Ashforth (2011) revealed that external organizational images influence the behavior of employees. So, when employees come across an image of their organization, they get motivated to play their role as a stakeholder and strive to relate it with their own identity (Scott and Lane, 2000) because they asses their self-worth based on the social standing of their organization (Tyler, 1999).

The study supported that the pride associated with the endorsement-linked recognition of their organization can help employees to strongly identify with their organization, which is in line with the findings of previous studies (Smidts et al., 2001; Fuller et al., 2006; Bartels et al., 2007; Celsi and Gilly, 2010; Khan et al., 2013). The employee perspective of SIT states that employees use the social status of their organization to guide them in estimating their self-worth (Tyler, 1999). Thus, individuals try to identify with organizations with prestigious standings because membership in a prestigious organization increases their self-esteem and fulfills the need for self-enhancement.

Hence, the study proved that brand orientation develops employees’ attitudes toward celebrity endorsement. Internal marketing theories state that internal association and interactions are important for employees’ involvement, shaping their positive attitudes and motivating them to implement profitable corporate programs (Saleem and Iglesias, 2016). By developing the employees’ attitude toward the celebrity endorser and his/her effectiveness, the employer could increase their identification with their organization. Furthermore, marketers and advertisers need to understand the importance of brand orientation on employees’ organization supportive behaviors toward organizations’ programs and policies, especially policies related to external marketing communication. The corporate internal communication process needs to be planned to ensure the product/service knowledge is constant and focuses on promise accuracy. The relevance of marketing strategies for employees with corporate values could strengthen their organizational identification.

## Conclusion

Employees are considered as one of the primary stakeholders, so their concerns toward the celebrity endorser should be examined while planning IMC techniques. Employees have extensive knowledge of their organization and use it while evaluating the effectiveness of celebrity endorsers. Therefore, marketers and advertisers should hire only celebrities congruent with organizational values. The celebrities should understand that they need to portray only the actual values of the employees and organization.

This research studied the effect of celebrity value congruence and employee portrayal accuracy on employees’ attitudes toward endorsement effectiveness. However, there is a need to study more about the employees’ attitudes toward celebrity endorsers. First, employees’ general and specific attitudes toward celebrities should be studied to understand how employees’ attitudes toward a celebrity could facilitate them to identify with the organization and its endorsement campaign. Second, the effect of other organizational factors, including human resources practices, working environment, and methods of service provisions, could be studied to evaluate employees’ reactions toward celebrity endorse. The internal communication process should be planned to ensure the provision of constant and detailed information to the employees of an organization. Also, the relevance of marketing activities with employees and organization values can strengthen their identification with their organization, enhance their commitment, and motivate them to adopt more supportive behavior. The study recommends that advertisers and marketers hire a celebrity endorser that matches the organization’s values so that employees may relate to it. Marketers must motivate committed organization followers to enhance their beliefs about the organizational policies. Employees’ internal communication should support positive and strong beliefs about the organization’s marketing initiatives.

As the employees were exposed to actual celebrity endorsers and endorsement advertisements of the organization, we might have missed the effects of controlled experimental research, which could control any bias toward celebrity/endorsement advertisement. The study evaluates the effect of employees’ attitudes toward celebrity endorsers on their identification with their organization; future research should be conducted to study the effects on different performance outcomes such as employee customer focus. Another limitation of this study is the limited sample size that might not represent the population, so further studies with larger sample sizes might be conducted to have a more accurate representation. Future research could also use multiple-level data from consumers and employees to analyze the impact of endorser effectiveness and identification.

## Data availability statement

The raw data supporting the conclusions of this article will be made available by the authors, without undue reservation.

## Author contributions

All authors listed have made a substantial, direct, and intellectual contribution to the work, and approved it for publication.
